# Burden and characteristics of HIV infection among female sex workers in Kampala, Uganda – a respondent-driven sampling survey

**DOI:** 10.1186/s12889-017-4428-z

**Published:** 2017-06-10

**Authors:** Wolfgang Hladik, Andrew L. Baughman, David Serwadda, Jordan W. Tappero, Rachel Kwezi, Namakula D. Nakato, Joseph Barker

**Affiliations:** 1 Division of Global HIV and TB, Center for Global Health, Centers for Disease Control and Prevention, 1600 Clifton Rd, Atlanta, GA 30333 USA; 2 Makerere University College of Health Sciences, School of Public Health, Kampala, Uganda; 3 Division of Global Health Protection, Center for Global Health, Centers for Disease Control and Prevention, Atlanta, GA USA; 4 WONETHA - Women’s Organisation Network for Human Rights Advocacy, Kampala, Uganda

**Keywords:** Female sex workers, HIV, STI, Kampala, Uganda, RDS, Respondent-driven sampling

## Abstract

**Background:**

Sex workers in Uganda are at significant risk for HIV infection. We characterized the HIV epidemic among Kampala female sex workers (FSW).

**Methods:**

We used respondent-driven sampling to sample FSW aged 15+ years who reported having sold sex to men in the preceding 30 days; collected data through audio-computer assisted self-interviews, and tested blood, vaginal and rectal swabs for HIV, syphilis, *neisseria gonorrhea, chlamydia trachomatis, and trichomonas vaginalis*.

**Results:**

A total of 942 FSW were enrolled from June 2008 through April 2009. The overall estimated HIV prevalence was 33% (95% confidence intervals [CI] 30%-37%) and among FSW 25 years or older was 44%. HIV infection is associated with low levels of schooling, having no other work, never having tested for HIV, self-reported genital ulcers or sores, and testing positive for *neisseria gonorrhea* or any sexually transmitted infections (STI). Two thirds (65%) of commercial sex acts reportedly were protected by condoms; one in five (19%) FSW reported having had anal sex. Gender-based violence was frequent; 34% reported having been raped and 24% reported having been beaten by clients in the preceding 30 days.

**Conclusions:**

One in three FSW in Kampala is HIV-infected, suggesting a severe HIV epidemic in this population. Intensified interventions are warranted to increase condom use, HIV testing, STI screening, as well as antiretroviral treatment and pre-exposure prophylaxis along with measures to overcome gender-based violence.

## Background

By the end of 2014, there were near 37 million people living with HIV globally, with more than 5000 new infections per day the majority of which occur in sub-Saharan Africa [1]. UNAIDS has put forward a bold vision to end the global HIV epidemic by 2030, [2] beginning by first meeting the 90-90-90 “fast-track” targets by 2020.^1^ These achievements are only possible by implementing comprehensive packages of prevention and HIV care services for all populations, especially those with highest HIV burden, incidence, and transmission rates.

Key populations, including sex workers, are critical to controlling the HIV pandemic. A recent review estimated that female sex workers (FSW) are 13.5 times more likely to be HIV infected than other women [3]. Sex workers require tailored public health interventions that address critical gaps in HIV prevention, HIV testing, case-finding, linkage, retention, and adherence to antiretroviral therapy. Although encouraging progress has been made in stabilizing HIV prevalence and promoting condom use among sex workers in some locations, substantially greater gains will be needed to reduce the rate of sexual HIV transmission among sex workers and their partners by 2020 [4]. FSW are at increased risk for HIV due to frequent sex with numerous clients, inconsistent condom use, anal sex, and drug use, as well as violence, stigma and discrimination, and impeded access to HIV services [5–7]. An estimated 15% of HIV infections worldwide may be due to sex work, with sub-Saharan Africa having the highest attributable fraction (17.8%) [8].

Uganda faces a generalized epidemic with an estimated adult HIV prevalence of 7.3% [9] and an HIV incidence that remains stubbornly stable [4]. In Kampala, Uganda’s capital, a cohort of high-risk women, most of whom reported engaging in selling sex, had a baseline HIV prevalence of 37% [10]. Sex work in Uganda is illegal and likely contributes disproportionally to the overall burden of disease; indeed, a Modes of Transmission study estimated that sex work-related HIV transmissions may account for 10% of incident HV infections [11].

As a socially hidden population, sex workers are often highly mobile, transitioning in and out of sex work as dictated by economic needs. Surveys using proper sampling designs for such populations are difficult and challenging, and thus convenience sampling designs are commonly used. We report here on the first bio-behavioral survey undertaken among FSW in Kampala that used a rigorous sampling design and thus generated population estimates for this important high-risk group. Our data collection and analysis was based on a conceptual framework in which social factors influence sexual and drug use behaviors that in turn affect HIV infection risk. The survey’s objectives were to: (1) estimate the burden of HIV and other sexually transmitted infections (STI), (2) estimate the burden of self-reported key behavioral measures relevant for HIV and STI control in this population, and (3) investigate risk factors associated with HIV infection.

## Methods

### Survey design

We conducted a cross-sectional biobehavioral survey among FSW in Kampala, Uganda, using respondent-driven sampling (RDS). Pre-survey formative research with eight key informants, including government officials, sex workers, and previous survey investigators using a qualitative interview guide suggested that RDS should be favored over venue-based sampling and informed seed identification, social connectedness, compensation for survey participation, as well as other survey procedures.

### Study population

Survey eligibility criteria included female sex, age ≥ 15 years, residence in greater Kampala, and self-reported selling of sex to one or more men during the 30 days preceding survey enrollment. Candidate recruits who received their coupons from strangers were excluded, as one of the functional RDS assumptions includes that recruiter and recruit know one another.

### Sampling

RDS methodology is well described elsewhere [12–14] and is commonly used to sample socially hidden populations. Sampling began with nine seeds and recruits received three coupons each as well as a brief training for peer referral. Recruits would give a coupon to a peer; only by presenting a coupon one could enroll in the survey. Due to a high coupon redemption rate, the number of coupons was quickly reduced to two coupons per recruits for most of the sampling period and further reduced to one towards the end of the survey. Recruits presented their coupons at the single survey office in downtown Kampala. Survey data were collected between June 2008 and April 2009.

**Fig. 1:**
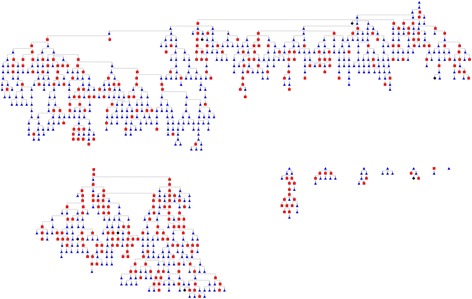
Diagram of the recruitment chains produced by yEd software (version 3.10.2). For the 949 nodes (FSW), 323 had a positive HIV test result (*red* square), 619 had a negative result (*blue* triangle), and 7 had a missing value (*black* circle)

In total, 2120 coupons were issued and 1100 (52%) were redeemed; an additional 40 coupons were deemed invalid. Of the 1100 candidate recruits, 949 (86%) were considered eligible, consented, and enrolled. Of these, 942 (99%) were interviewed and received HIV testing. The number of waves per seed varied between 1 and 25; equilibrium (i.e., the point in sampling when the distribution of a given characteristic stabilizes) for both HIV infection and age was reached after wave 2. Figure 1 displays the sampling tree; Table 1 shows the recruitment matrix by HIV status and age group.

**Table 1:** Recruiter-recruit matrix by HIV status and age group

		Recruit	
	HIV status	Negative	Positive
Recruiter	Negative	407 (70%)	174 (30%)
	Positive	205 (58%)	147 (42%)
	Age	15-24 years	25+ years
	15-24 years	178 (51%)	172 (49%)
	25+ years	220 (38%)	363 (62%)

### Survey office procedures

After recruits’ coupons were verified, CD and MP3 players were used to inform recruits about specific interview terms used (such as the different types of sex, frequency of sex, partner types), peer recruitment, and other relevant survey information. Candidate participants were then screened for eligibility and consented face-to-face. Thereafter, a short computer-based tutorial about audio-computer-assisted self-interviewing (ACASI) preceded the actual ACASI; a small fraction who could not complete the ACASI were interviewed face-to-face. Following the interview, recruits underwent pre-test counseling for HIV and STI testing and had venous blood as well as vaginal and rectal swab specimens collected. At the end of the first visit, recruits received instructions and coupons for peer recruitment. Recruits were asked to return to the survey office two weeks later to receive post-test counseling for HIV and other test results, treatment for non-HIV associated STI, or referral letters for HIV care/treatment providers in case of HIV-positive results.

### Data measures

In addition to basic demographic data, our main data variables of interest included lifetime sexual characteristics, sexual experiences in the last 30 days, sexual violence, self-reported alcohol and drug use, sexually transmitted disease (STD) symptoms, access to HIV services, and HIV-related perceptions. We probed blackmail through the question “*Have you ever been blackmailed by someone because you sell sex?*”, and knowledge on transmission risk by mode of intercourse through the question “*What kind of sex do you think is more dangerous to get HIV?*” We probed alcohol consumption as having ever drunk alcohol, probed the frequency of alcohol drinking in the last 30 days and prior to the last sex act, and defined a drink of alcohol as “*one bottle of beer, one glass of wine, or one shot of whisky or waragi.*”

### Laboratory measures

Specimens were transported daily to the laboratory. Laboratory testing was performed at the STD Reference Laboratory based at Mulago Hospital, Kampala. Testing for HIV-1 antibodies was performed in parallel using Vironostika® HIV Uniform II plus O2 (bioMeriéux, Marcy l’Etoile, France) and Murex® HIV Ag/Ab Combination (Abbott Laboratories, Abbott Park, Illinois, U.S.A.). Serological testing for *Treponema pallidum* (TP) was performed with anti-syphilis IgG ELISA (Biotec Laboratories, Suffolk, UK) and, if reactive, the Rapid Plasma Reagin Syfacard-R Test (Murex Biotech, Dartford, UK) to detect current TP infection. Vaginal and rectal swabs were tested for *Neisseria gonorrhea* (NG) and *Chlamydia trachomatis* (CT) DNA using Cobas Amplicor or Amplicor PCR (Roche Diagnostics, Branchburg, New Jersey, U.S.A.). Using microscopy, we evaluated vaginal swabs for the presence of bacterial vaginosis, and *Trichomonas vaginalis* utilizing InPouch (BioMed Diagnostics, Inc., White City, Oregon, USA).

### Data management and analysis

For sample size calculations, we assumed an HIV prevalence of 25.2%, approximately twice that of urban Ugandan women in general [15] (no probability-based previous survey estimates for FSW in Kampala were available), and a design effect of 2 [16], and determined that a minimum target sample size of approximately 600 would yield acceptable 95% confidence intervals (CI). Interview data were cleaned using Statistical Analysis Software - SAS v9.3 (SAS Institute, Cary, North Carolina). To account for social network size and recruitment patterns, we calculated individualized weights using RDSAT version 7.1.38 software (www.respondentdrivensampling.org). For categorical variables the RDS-based weights were applied to the data and each FSW was treated as a primary sampling unit using SAS-callable SUDAAN software [17]. For several continuous variables, we calculated the median (interquartile range, IQR) using unweighted data.

Chi-squared tests of independence between each characteristic and the HIV outcome were performed. In addition, we calculated prevalence ratios and 95% CI [18]. Because numerous characteristics of FSW were associated with HIV infection, we used a hierarchical conceptual framework for building a multivariable model for HIV infection [19]. First, we included sociodemographic factors associated with HIV infection at a level of *P* < 0.2 in bivariate analysis in a backward elimination model selection procedure and retained factors in the model that were independently associated with HIV infection (*P* < 0.05). Next, we considered this core set of sociodemographic factors plus behavioral factors that were associated with HIV infection at a level of *P* < 0.2 in bivariate analysis in a second backward elimination model selection procedure. The final set of sociodemographic and behavioral factors independently associated with HIV infection were included in a separate multivariable model for each of the other sexually transmitted infections.

### Human subjects considerations

The survey was conducted anonymously; we obtained verbal informed consent but collected no personal identifiers. Recruits had their fingerprints scanned (images were not stored) to generate unique alphanumeric codes and facilitate linking visits, detecting duplicate enrollment attempts or coupons that were issued to other recruits. Several different surveys were carried out at the same time using the same survey office, thus masking the group identity of respondents visiting the survey office. We compensated recruits for their time and transport costs (US $3.00), and, at the return visit, recruitment efforts (US $1.00 per successfully recruited eligible peer). For FSW under the age of 18 who indicated that they were forced into sex work, procedures were in place to refer such persons to relevant service providers.

## Results

### Population characteristics

Table 2 displays select characteristics of FSW in Kampala. Almost all (95%) were of Ugandan nationality and 44% were 15–24 years old (median age: 26 years). Most (83%) FSW have ever been to school, and 51% had completed at least 7 years of schooling, equivalent to primary school education. Most (66%) FSW had ever been married and most had had at least one pregnancy in their lifetime (91%). The median number of pregnancies was 3 (IQR: 1–4), and 25% had ever had an abortion. Family planning use was relatively common, with 67% using a modern method, including injectables (34%) and oral contraceptives (31%).

**Table 2:** Characteristics of female sex workers in Kampala, Uganda, 2008–09, *N* = 942

Characteristic^a^	*n*	Sample %	Weighted % (95% CI)
Age, years			
15–19	109	11.6	12.6 (9.9–15.6)
20–24	292	31.0	32.4 (28.4–36.1)
25–29	248	26.3	25.5 (22.3–28.8)
30+	293	31.1	29.6 (25.8–33.6)
Nationality			
Ugandan	897	95.7	95.4 (93.7–96.9)
Not Ugandan	40	4.3	4.6 (3.0–6.3)
Schooling, years			
None	149	16.2	17.3 (14.3–20.3)
1–3	103	11.2	12.0 (9.1–14.7)
4–6	185	20.1	19.7 (16.8–23.0)
7+	482	52.6	51.0 (46.9–55.4)
Marital status			
Never married	301	32.2	33.8 (29.9–37.9)
Cohabitating	210	22.5	22.3 (19.3–25.6)
Married (mono)	55	5.9	5.9 (4.2–7.5)
Married (poly)	141	15.1	13.9 (11.6–16.5)
Div/sep/wid^b^	228	24.4	24.1 (20.7–27.4)
Ever been married			
Yes	634	67.8	66.2 (62.2–70.1)
No	301	32.2	33.8 (30.0–37.8)
Pregnancies in life, number			
0	74	8.1	9.4 (6.7–12.6)
1	174	19.0	21.3 (17.9–24.5)
2	173	18.9	18.3 (15.9–21.8)
3	164	17.9	17.4 (14.5–19.8)
4	134	14.6	14.6 (11.8–17.1)
5+	196	21.4	19.1 (16.1–22.3)
Ever aborted a pregnancy			
Yes	257	27.5	24.9 (21.5–28.6)
No^c^	679	72.5	75.1 (71.5–78.5)
Use of modern family planning^e^			
Yes	624	67.0	64.7 (60.3–68.3)
No	307	33.0	35.3 (31.7–39.7)
Steady male sex partners last 30 days, number			
0	168	18.2	18.5 (15.4–21.8)
1	288	31.1	31.1 (27.2–35.0)
2	194	21.0	20.5 (17.2–23.3)
3+	275	29.7	29.9 (26.5–34.0)
Sex work main income			
Yes	808	88.4	87.1 (84.5–89.8)
No	106	11.6	12.9 (10.2–15.4)
Occupation other than sex work			
No other work	557	59.6	57.2 (53.7–61.3)
Other	113	12.1	13.5 (10.8–16.3)
Self-employed	148	15.8	16.3 (13.5–19.1)
Restaurant/bar	116	12.4	13.1 (10.1–15.7)

^a^For each characteristic, the total sample size differs from 942 due to missing information

^b^Divorced, separated, widowed

^c^Includes those who have never been pregnant

^d^For each characteristic, the total sample size differs from 942 due to missing information

^e^Oral contraceptives, intra-uterine device, or injection (depot provera)

### Sex work characteristics

The median duration of sex work at time of survey participation was 3 years (IQR: 2–6); the median age of first selling sex was 22 years (IQR 18–26). For most (57%), sex work was the only available income. The median fee for sex was approximately US $3 (IQR $2–5). FSW meet their clients mostly (71%) on the street, and have sex with them mostly at their or someone else’s home (88%). Almost half (46%) of FSW have ever been forced to have sex, and 34% reported being raped in the last 30 days. Out of the times being raped (defined as being forced or threatened to be hurt to have vaginal or anal sex) in the last 30 days, 26% were by a customer, and 16% by security personnel or police. Some of these incidents were reported to the police (17%) and health care was sought (18%). Beatings by clients in the last 30 days were reported by 24% of FSW. Blackmail due to knowledge by someone of their sex work was low (16%). A small proportion (3%) ever sold sex to women. In the preceding 12 months, 14% of FSW had sold sex outside of Kampala.

### HIV-related risk behaviors and perceptions

During the last sex act with a male client, two thirds (65%) used a condom and 14% used a lubricant; 11% reported that they were forced to have sex. Additionally, 9% reported to have taken illicit drugs prior to that sex act and half (49%) said they had drunk alcohol.

Lifetime alcohol use was reported by 77%, with 29% had been drinking every day over the last 30 days with an average number of 2.9 drinks the last time they had consumed alcohol. Drug use, including sniffing glue or petrol, or consuming marijuana, khat (a plant-based mild stimulant), cocaine, or other drugs was reported by 24%. A lifetime history of injecting drug use was reported by 4%. Half (49%) of FSW had sex with 8 or more men (including intimate partners) over the preceding 30 days. The median proportion of condom use for all male partners in the last 30 days was 60% (IQR 24–88%), with 18% of FSW having used condoms 100% of the time. Approximately one fifth (19%) of sex workers ever had anal sex and 15% had anal sex in the last 30 days; among these, the median number of anal sex acts in the last 30 days was 3. Among FSW who ever had anal sex, 30% felt it was less important to use condoms for anal sex compared to vaginal sex. Only 15% thought anal sex put them at higher risk of contracting HIV than vaginal sex.

### HIV services uptake

The majority (53%) of FSW had previously tested for HIV, and 16% had tested in the preceding 12 months. Among those who ever tested for HIV, 92% reportedly tested negative and 8% told their HIV (negative or positive) status to their sex partners; 89% did not reveal their HIV status to anyone. Of those who reported having tested negative, 16% were found to be HIV-positive. Among those who were known HIV-positive, 52% said that they were on anti-retroviral treatment (ART).

### HIV infection

Of 942 participants, 323 tested HIV-positive, yielding a weighted HIV prevalence of 33.0% (95% CI: 29.7%–36.6%). Table 3 shows the distribution of HIV infection by select characteristics. In this bivariate analysis, factors associated with HIV serostatus included age, marital status, number of pregnancies, self-reported HIV status, duration of selling sex, self-reported clinical signs and symptoms of STIs, and a history of previous HIV testing.

**Table 3:** Association between sociodemographic or behavioral characteristics of female sex workers and HIV infection in Kampala, Uganda

Characteristic^d^	n	HIV+	Weighted %	*P*-value^e^	Prevalence Ratio (95% CI)
Age, years					
15–19	109	11	10.8	<0.01	1.00 (Referent)
20–24	291	65	21.9		2.03 (1.04–3.95)
25–29	246	88	35.9		3.33 (1.74–6.40)
30+	293	157	50.7		4.71 (2.50–8.89)
Nationality					
Ugandan	894	304	33.0	0.99	1.00 (Referent)
Not Ugandan	40	16	33.1		1.00 (0.61–1.65)
Religion					
Catholic	362	131	35.4	0.41	1.00 (Referent)
Protestant	262	86	30.9		0.87 (0.68–1.13)
Born Again	47	17	37.0		1.05 (0.64–1.71)
Moslim	232	71	28.5		0.81 (0.61–1.07)
Other	30	13	42.9		1.21 (0.74–1.98)
Schooling, years					
0–3	251	107	42.1	<0.01	1.00 (Referent)
4–6	185	70	36.6		0.87 (0.66–1.14)
7+	482	137	26.4		0.63 (0.49–0.80)
Sex work main income					
Yes	806	286	35.1	<0.01	1.68 (1.13–2.51)
No	106	28	20.9		1.00 (Referent)
Occupation other than sex work					
No other work	557	201	36.9	<0.01	1.00 (Referent)
Other	112	26	19.2		0.52 (0.34–0.79)
Self-employed	147	53	32.3		0.88 (0.66–1.17)
Restaurant/bar	115	36	26.7		0.72 (0.50–1.05)
Marital status					
Never married	300	71	23.4	<0.01	1.02 (0.57–1.83)
Cohabitating	210	83	37.8		1.64 (0.93–2.91)
Married (mono)	53	14	23.0		1.00 (Referent)
Married (poly)	141	46	36.5		1.59 (0.88–2.86)
Div/sep/wid	228	105	41.9		1.84 (1.04–3.20)
Pregnancies in life, number					
0	74	4	3.6	<0.01	1.00 (Referent)
1	173	36	21.0		5.88 (2.00–17.3)
2	173	61	33.4		9.34 (3.26–26.8)
3	162	65	44.8		12.5 (4.40–35.5)
4	134	60	46.5		13.0 (4.56–36.9)
5+	196	86	38.7		10.8 (3.81–30.7)
Ever aborted					
Yes	231	60	27.4	0.07	0.79 (0.60–1.03)
No^h^	702	260	34.7		1.00 (Referent)
Steady male sex partners last 30 days, number					
0	167	68	40.4	0.18	1.00 (Referent)
1	288	105	33.3		0.82 (0.62–1.09)
2	192	57	28.0		0.69 (0.50–0.96)
3+	275	86	31.7		0.79 (0.59–1.05)
Alcohol before last sex					
Yes	480	173	35.0	0.25	1.13 (0.92–1.40)
No	455	147	31.0		1.00 (Referent)
Drugs for pleasure					
Yes	217	71	34.0	0.71	1.05 (0.82–1.34)
No	708	244	32.4		1.00 (Referent)
Inject drugs					
Yes	42	16	40.6	0.38	1.24 (0.80–1.91)
No^i^	886	303	32.8		1.00 (Referent)
Years selling sex, number					
< 1	13	4	32.7	0.02	1.00 (Referent)
1	148	34	21.3		0.65 (0.26–1.62)
2	179	58	31.4		0.96 (0.40–2.31)
3	177	62	32.3		0.99 (0.41–2.36)
4	74	23	33.6		1.03 (0.41–2.57)
5+	335	136	39.9		1.22 (0.52–2.87)
Condom use during last sex with a male client					
Yes	619	208	32.0	0.50	0.93 (0.75–1.15)
No	315	111	34.6		1.00 (Referent)
Ever used condom					
Yes	872	301	33.3	0.54	1.00 (Referent)
No	56	16	28.9		0.87 (0.53–1.41)
Ever used lubricant					
Yes	282	90	30.8	0.44	1.00 (Referent)
No	648	228	33.6		1.09 (0.87–1.38)
Violence due to selling sex					
Yes	433	163	35.9	0.13	1.17 (0.95–1.45)
No	498	155	30.5		1.00 (Referent)
Raped in last 30 days, number					
0^c^	619	206	32.4	0.38	1.00 (Referent)
1	93	36	36.2		1.12 (0.77–1.62)
2	81	25	27.3		0.84 (0.57–1.26)
3	43	20	47.9		1.48 (1.02–2.15)
4+	91	28	31.6		0.97 (0.68–1.40)
Raped by customer in last 30 days, number					
0^j^	682	228	32.4	0.94	1.00 (Referent)
1	124	40	30.0		0.92 (0.65–1.32)
2	71	26	36.1		1.11 (0.77–1.60)
3	25	9	31.9		0.98 (0.51–1.91)
4+	23	8	38.4		1.18 (0.64–2.20)
Sex partners, total number in last 30 days					
0–12	221	71	29.9	0.59	1.00 (Referent)
13–24	234	75	31.8		1.06 (0.78–1.45)
25–58	248	87	36.2		1.21 (0.90–1.62)
59+	236	88	34.4		1.15 (0.85–1.55)
Male partners in life, number					
1–24	201	58	25.1	0.08	1.00 (Referent)
25–64	212	70	34.6		1.38 (0.98–1.93)
65–194	211	76	33.1		1.32 (0.94–1.86)
195+	209	73	38.3		1.53 (1.09–2.13)
Vaginal discharge/burning in last 12 months					
Yes	644	233	35.6	0.03	1.31 (1.02–1.68)
No	280	82	27.2		1.00 (Referent)
Genital ulcer/sore in last 12 months					
Yes	520	211	39.6	<0.01	1.58 (1.26–1.98)
No	409	107	25.0		1.00 (Referent)
Anal ulcer/sore in last 12 months					
Yes	204	82	41.0	0.03	1.32 (1.05–1.65)
No	717	235	31.1		1.00 (Referent)
Anal discharge in last 12 months					
Yes	124	42	33.8	0.79	1.04 (0.77–1.41)
No	791	270	32.4		1.00 (Referent)
Anal warts in last 12 months					
Yes	132	65	49.7	<0.01	1.65 (1.32–2.08)
No	783	247	30.0		1.00 (Referent)
Ever had anal sex					
Yes	171	62	36.2	0.38	1.12 (0.87–1.45)
No	758	256	32.2		1.00 (Referent)
Ever had HIV test					
Yes	503	120	23.0	<0.01	1.00 (Referent)
No	430	199	44.6		1.93 (1.55–2.41)
Had steady male sex partners last 30 days					
Yes	755	248	31.4	0.06	0.78 (0.61–0.99)
No	167	68	40.4		1.00 (Referent)

^d^For each characteristic, the total sample size differs from 942 due to missing information

^e^Chi-squared test of independence between the characteristic and HIV positivity

^f^For each characteristic, the total sample size differs from 942 due to missing information

^g^Chi-squared test of independence between the characteristic and HIV positivity

^h^Includes those who have never been pregnant

^i^Inlcudes those who have never used drugs

^j^Includes those who have never been raped

In the adjusted analysis (Table 4), factors independently associated with HIV positivity included increasing age, fewer years of schooling, having no other work than sex work, increasing numbers of pregnancies, never having had an abortion, never having had an HIV test, and a history of STI-related symptoms in the last 12 months. Further, testing positive for *N. gonorrhea* (vaginal or rectal), and testing positive for any STI was associated with being HIV-infected (Table 5).

**Table 4:** Factors associated with HIV infection among female sex workers, Kampala, Uganda, 2008–09

Characteristic	n	HIV+	Weighted %	Adjusted PR (95% CI)^a^
Age, years				
15–19	109	11	10.8	1.00 (Referent)
20–24	291	65	21.9	1.27 (0.74–2.19)
25–29	246	88	35.9	1.64 (0.92–2.90)
30+	293	157	50.7	2.13 (1.19–3.80)
Schooling, years				
1–3	251	107	42.1	1.00 (Referent)
4–6	185	70	36.6	0.82 (0.63–1.07)
7+	482	137	26.4	0.67 (0.54–0.84)
Occupation other than sex work				
No other work	557	201	36.9	1.00 (Referent)
Other	112	26	19.2	0.55 (0.37–0.81)
Self-employed	147	53	32.3	0.74 (0.55–0.99)
Restaurant/bar	115	36	26.7	0.80 (0.60–1.08)
Pregnancies in life, number				
0	74	4	3.6	1.00 (Referent)
1	173	36	21.0	7.24 (2.14–24.5)
2	173	61	33.4	9.43 (2.75–32.3)
3	162	65	44.8	11.7 (3.38–40.7)
4	134	60	46.5	10.8 (3.06–37.9)
5+	196	86	38.7	8.23 (2.29–29.6)
Ever aborted				
Yes	231	60	27.4	0.76 (0.59–0.97)
No	702	260	34.7	1.00 (Referent)
Genital ulcer/sore in last 12 months				
Yes	520	211	39.6	1.39 (1.13–1.71)
No	409	107	25.0	1.00 (Referent)
Anal warts in last 12 months				
Yes	132	65	49.7	1.43 (1.14–1.80)
No	783	247	30.0	1.00 (Referent)
Ever had HIV test				
Yes	503	120	23.0	1.00 (Referent)
No	430	199	44.6	1.71 (1.39–2.10)

NOTE: PR, prevalence ratio

^a^The multivariable model included all of the factors in this table. The sample size for the model was 861 female sex workers

**Table 5:** Association between other sexually transmitted infections and HIV infection among female sex workers, Kampala, Uganda, 2008–09

STI^a^	n	HIV+	Weighted %	Adjusted PR (95% CI)^b^
Syphilis				
Positive	193	81	38.9	1.22 (0.98–1.52)
Negative	745	239	31.2	1.00 (Referent)
Chlamydia trachomatis vaginal				
Positive	39	8	16.9	0.90 (0.54–1.51)
Negative	683	239	33.9	1.00 (Referent)
Chlamydia trachomatis rectal				
Positive	22	4	13.2	0.80 (0.30–2.10)
Negative	706	246	33.8	1.00 (Referent)
Neisseria gonorrhoeae vaginal				
Positive	55	29	49.2	1.64 (1.27–2.10)
Negative	667	218	31.8	1.00 (Referent)
Neisseria gonorrhoeae rectal				
Positive	27	16	58.9	1.96 (1.44–2.66)
Negative	701	234	32.4	1.00 (Referent)
Trichomonas vaginalis				
Positive	73	26	32.5	1.00 (0.70–1.42)
Negative	773	263	33.3	1.00 (Referent)
Bacterial vaginosis				
Positive	318	128	39.9	1.19 (0.98–1.46)
Negative	526	160	29.0	1.00 (Referent)
Any STI				
Positive	511	197	37.2	1.28 (1.05–1.56)
Negative	431	126	27.9	1.00 (Referent)

NOTE: STI, sexually transmitted infection; PR, prevalence ratio

^a^For each STI, the total sample size differs from 942 due to missing information

^b^Adjusted for age, schooling, occupation other than sex work, number of pregnancies in life, ever aborted, genital ulcer/sore in last 12 months, anal warts in last 12 months, and ever had an HIV test

## Discussion

### Main finding

We report here on the first RDS survey among FSW in Kampala, Uganda, using a sampling design that allows us to generate population estimates. The estimated HIV prevalence of 33% suggests an alarming HIV burden among FSW, more than three times that among Kampala women in general (9.5% [9]). This estimate is similar to that reported as the baseline in a cohort study in Kampala conducted at approximately the same time [10]. Our population-based survey, using a probability sample, can serve as a baseline estimate for subsequent surveys in this population in Kampala. The median duration of sex work at time of survey participation was just 3 years. The much higher HIV prevalence in FSW compared to Kampala women in general together with the relatively short median duration of sex work would suggest a very high HIV incidence, although our cross-sectional survey design did not allow us to estimate HIV incidence. In adjusted analysis, increasing age, low levels of schooling, having no other work, lack of ever having tested for HIV, self-reported genital ulcers or sores, as well as testing positive for *N. gonorrhea* or any STI were associated with being HIV-infected.

### Limitations and strengths

Our survey’s limitations mostly relate to the RDS design, which may not have been able to reach isolated sex workers with few or no peer connections. Also, as in all surveys, all behavioral parameters evaluated here are based on self-reported interview data, which are subject to reporting bias. The use of ACASI in our survey likely can be seen as a strength, as it facilitates the reporting of sensitive behaviors [20–22], augmented by the objective biomarker collection using blood as well as vaginal and rectal swabs. The survey’s main strength likely lies in its sampling design using RDS. Previous FSW surveys used convenience sampling [10], making this the first representative sex worker survey in Kampala.

### Implications for policy and program

Our survey’s findings call for targeted strengthening of programs promoting condom use (among both sex workers and clients), regular HIV and STI testing, pre-exposure prophylaxis, and prompt treatment among FSW. Although almost all FSW reported some condom use, about one third did not use condoms at their last commercial sex act, an estimate that may further suffer from reporting bias despite the ACASI-based interview format. Consistent and correct condom use is highly effective against HIV and STI transmission and needs to be a cornerstone in sex work–based HIV control efforts. [5] Only one in seven Kampala FSW had tested for HIV in the preceding 12 months. UN and donor agencies recommend universal access to comprehensive HIV services for sex workers as a central component of policies related to sex work. [5] Regular HIV testing, at least annually, is warranted for a population at such high risk for HIV infection. [5] Our observation of self-reported anal sex by approximately one in five sex workers suggests that prevention and counseling services in Uganda need to address this high-risk behavior. Barriers to HIV testing, counseling, and related health care for sex workers, described both in Uganda and elsewhere in Africa and fueled by criminalization and stigma [23], need to be overcome.

Screening for STI is not widely available for sex workers in Uganda, is often based on syndromic management and rarely includes management of rectal STI. Our survey confirms the presence of common STI among FSW based on both self-report of genital and anal ulcers and biological testing for trichomoniasis, syphilis, chlamydia, and gonorrhea. In addition to the disease burden they inflict by themselves, STI increase the risk of HIV transmission. [24, 25] Regular screening for STI or – in its absence – presumptive treatment as recommended by UNAIDS [5] should therefore be considered through public or non-profit programs tailored for FSW in order to minimize exposure to stigma, along with increased investment in laboratory testing capacity for common STI.

Added to the low rate of HIV testing is the equally low reported rate of disclosing their HIV test result to their sex partners and the uptake of ART among HIV-positive FSW. Recent guidance by the World Health Organization [26] suggests that all FSW, and indeed all key populations, should take antiretrovirals either for treatment (HIV-infected, regardless of CD4+ T-cell count) or for pre-exposure prophylaxis (HIV-uninfected). This implies not only frequent HIV testing and self-disclosure by sex workers but also increased efforts by health care providers to facilitate access, reduce stigma, and counsel for HIV status disclosure to their partners.

Of grave concern is the frequent experience of gender-based violence (GBV) among FSW, including rape and beatings. UNAIDS’ vision of zero new HIV infections, zero discrimination, and zero AIDS-related deaths also includes zero tolerance for GBV [27]. GBV against FSW are well documented in Uganda [28] and elsewhere in the region. [15, 29] Decriminalization of sex work as well as programs to eliminate and to provide redress for violence and discrimination should be put in place.

The high burden of HIV disease among sex workers in Kampala suggests that the goal of 90%-90%-90% targets for testing, treatment and viral load suppression and the vision of an AIDS-free generation will not be achieved unless the HIV epidemic in this key population is contained. In addition to the use of antiretrovirals, recommendations for controlling sex work–related HIV transmission include decriminalization of sex work, which may also reduce stigma and violence; improved access to HIV testing, STI screening, and other prevention and treatment services; as well as peer education and peer-led interventions such as condom negotiation skills and other health promotion measures [5, 30]. To achieve this, community-based sex worker organizations in Uganda need to be seen as essential partners in combating sex work–related HIV and STIs.

The effectiveness of HIV control and other public health measures in hidden populations such as sex workers requires regular monitoring of endpoints such as the incidence or prevalence of HIV, estimates of viral load suppression, and perhaps testing for antiretrovirals. In order to evaluate and inform control programs, population-based surveys using rigorous sampling designs need to be conducted regularly given the likely high HIV incidence, geographic and occupational mobility, and levels of access to and uptake of HIV services that may not mirror that in the general population.

## Conclusions

FSW in Kampala are at an extremely high risk for HIV infection and violence. To achieve the laudable and ambitious goal of zero new infections, key populations such as sex workers need to be included in all aspects of HIV prevention, care, and treatment programs. Uganda’s national AIDS control strategy needs to recognize the HIV epidemic in sex workers as a public health crisis that will not cede without intensified public health efforts.

## Abbreviations

ACASI: Audio computer assisted self interview
AIDS: Acquired immunodeficiency syndrome
ART: Antiretroviral therapy
CD: Compact disc
CDC: Centers for Disease Control and Prevention
CI: Confidence intervals
CT: Chlamydia trachomatis
FSW: Female sex worker(s)
GBV: Gender-based violence
HIV: Human immunodeficiency virus
IQR: Interquartile range
MP3: MPEG-1 and/or MPEG-2 Audio Layer III
NG: Neisseria gonorrhea
RDS: Respondent driven sampling
SAS: Statistical Analysis System
STD: Sexually transmitted diseases
STI: Sexually transmitted infections
TP: Treponema pallidum
UK: United Kingdom
UN: United Nations
UNAIDS: Joint United Nations Programme on AIDS
USA: United States of America

## Footnotes

1 By 2020, 90% of all people living with HIV will know their HIV status; 90% of all people with diagnosed HIV infection will receive sustained antiretroviral therapy; and 90% of all people receiving antiretroviral therapy will have viral suppression.
